# TAZ contributes to pulmonary fibrosis by activating profibrotic functions of lung fibroblasts

**DOI:** 10.1038/srep42595

**Published:** 2017-02-14

**Authors:** Satoshi Noguchi, Akira Saito, Yu Mikami, Hirokazu Urushiyama, Masafumi Horie, Hirotaka Matsuzaki, Hideyuki Takeshima, Kosuke Makita, Naoya Miyashita, Akihisa Mitani, Taisuke Jo, Yasuhiro Yamauchi, Yasuhiro Terasaki, Takahide Nagase

**Affiliations:** 1 Department of Respiratory Medicine, Graduate School of Medicine, The University of Tokyo, 7-3-1 Hongo, Bunkyo-ku, Tokyo 113-0033, Japan; 2 Division for Health Service Promotion, The University of Tokyo, 7-3-1 Hongo, Bunkyo-ku, Tokyo 113-0033, Japan; 3 Department of Clinical Laboratory, The University of Tokyo Hospital, 7-3-1 Hongo, Bunkyo-ku, Tokyo 113-8655, Japan; 4 Department of Analytic Human Pathology, Graduate School of Medicine, Nippon Medical School, 1-1-5 Sendagi, Bunkyo-ku, Tokyo 113-8602, Japan

## Abstract

Transcriptional coactivator with PDZ-binding motif (TAZ) regulates a variety of biological processes. Nuclear translocation and activation of TAZ are regulated by multiple mechanisms, including actin cytoskeleton and mechanical forces. TAZ is involved in lung alveolarization during lung development and *Taz*-heterozygous mice are resistant to bleomycin-induced lung fibrosis. In this study, we explored the roles of TAZ in the pathogenesis of idiopathic pulmonary fibrosis (IPF) through histological analyses of human lung tissues and cell culture experiments. TAZ was highly expressed in the fibroblastic foci of lungs from patients with IPF. TAZ controlled myofibroblast marker expression, proliferation, migration, and matrix contraction in cultured lung fibroblasts. Importantly, actin stress fibers and nuclear accumulation of TAZ were more evident when cultured on a stiff matrix, suggesting a feedback mechanism to accelerate fibrotic responses. Gene expression profiling revealed TAZ-mediated regulation of connective tissue growth factor (CTGF) and type I collagen. Clinical relevance of TAZ-regulated gene signature was further assessed using publicly available transcriptome data. These findings suggest that TAZ is involved in the pathogenesis of IPF through multifaceted effects on lung fibroblasts.

The Hippo pathway, which was originally identified in *Drosophila*, is an emerging signaling pathway that plays important roles regulating cell proliferation, differentiation, stem cell renewal, and tumorigenesis in mammals^1^. Transcriptional co-activator with PDZ-binding motif (TAZ) and its homolog, Yes-associated protein (YAP), are phosphorylated and inactivated by the Hippo pathway. Subcellular localization of TAZ/YAP is regulated by several mechanisms, such as cell polarity, cell junction proteins, and actin cytoskeleton. TAZ/YAP cooperates mainly with the TEA domain (TEAD) family of transcription factors after nuclear translocation to stimulate expression of genes that regulate cell proliferation, differentiation, and apoptosis^2^.

We previously determined the critical role of TAZ during lung development. *Taz*-knockout mice display abnormal alveolarization with enlarged airspaces resembling human pulmonary emphysema^3^. TAZ is expressed in the epithelium of developing lung and interacts with thyroid transcription factor-1 (TTF-1) in lung epithelial cells, suggesting an important role of TAZ in lung morphogenesis and homeostasis^4^,^5^. We also showed that TAZ is amplified in a subset of non-small cell lung cancer and higher expression predicts poor prognosis. Furthermore, TAZ contributes to tumorigenesis of lung cancer cells by controlling cell cycle and apoptosis^6^.

Idiopathic pulmonary fibrosis (IPF) is a progressive chronic interstitial lung disease with poor outcome and is characterized by the accumulation of fibroblasts and remodeling of extracellular matrix (ECM). Fibroblasts play central roles in fibrotic processes and contribute to histological features of IPF lung tissues, including clusters of proliferating fibroblasts called “fibroblastic foci”^7^. Fibroblasts in IPF lung tissues have distinct properties, such as a high capacity for ECM production, secretion of proteases, and contractile ability^8^. These activated fibroblasts are characterized by the expression of α-smooth muscle actin (α-SMA), and are called as myofibroblasts. It is assumed that myofibroblast differentiation is a key step in the development of fibrosis. Myofibroblasts aberrantly proliferate, evade apoptosis, and impede the resolution of fibrosis in IPF lung tissues^7^.

The physiological hallmarks of IPF include increased lung stiffness, which is measured by the pressure-volume relationship^9^. Moreover, a progressive decrease in vital capacity is observed to varying degrees and is linked to aggravated dyspnea and impaired quality of life. These features are associated with structural changes caused by remodeling of the ECM and contraction of fibroblasts, both of which contribute to tissue tension or stiffness.

We previously showed that *Taz*-heterozygous mice are resistant to bleomycin-induced lung fibrosis^3^. Bleomycin-treated *Taz*-heterozygous mice have lower tissue fibrosis scores, hydroxyproline content, and lung elastance. These results indicate that TAZ plays a significant role in lung fibrosis, but the cellular and molecular mechanisms have not been elucidated. Indeed, it remained undetermined which cell types express TAZ in the adult lung and how decreased TAZ leads to the attenuation of fibrosis.

In this study, we demonstrated that TAZ is activated in lung fibroblasts of patients with IPF and that it is functionally involved in the pathogenesis of IPF through its multifaceted effects on lung fibroblasts.

## Results

### TAZ is activated in the fibroblastic foci of lung specimens from patients with IPF

We first surveyed TAZ transcript levels in lung epithelial cells and fibroblasts, with reference to the ZENBU database, which provides gene expression profiles of human primary cells, tissues, and cancer cells derived from various tissues (http://fantom.gsc.riken.jp/zenbu/). We found relatively higher TAZ expression in lung fibroblasts compared to that in bronchial epithelial cells, small airway epithelial cells, and alveolar epithelial cells (Supplementary Fig. S1a). Furthermore, analysis of the publicly available microarray dataset, GSE40839 revealed that TAZ transcript levels are higher in lung fibroblasts derived from patients with IPF compared to those in normal control lung fibroblasts (Supplementary Fig. S1b)^10^.

**Figure 1:**
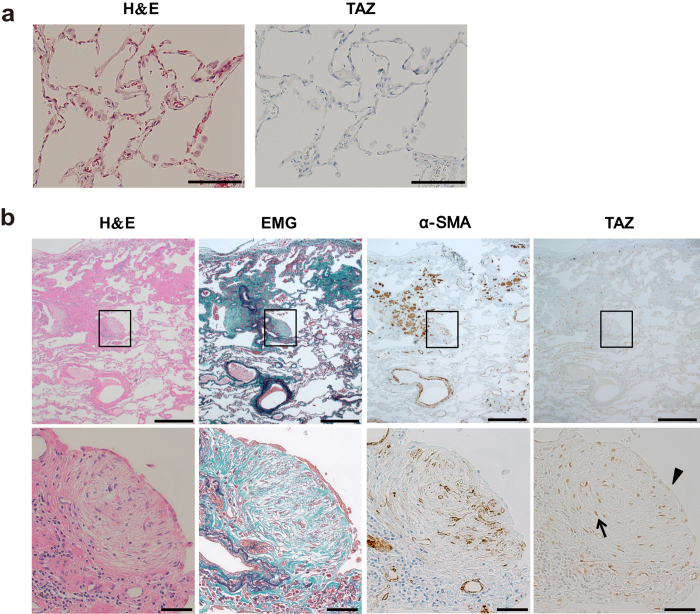
TAZ expression in the alveoli of normal lungs, and the fibroblastic foci in idiopathic pulmonary fibrosis (IPF). (**a**) Representative hematoxylin and eosin (H&E) image and TAZ immunohistochemistry (IHC) in the normal alveoli. TAZ immunoreactivity was nearly negative. Scale bar = 50 μm. (**b**) Representative H&E and Elastica Masson Goldner (EMG) staining, and IHC for α-smooth muscle actin (α-SMA) and TAZ in the fibroblastic foci of IPF lungs. Upper panel: low magnification. The fibroblastic foci were found in IPF lung tissues (black box). Scale bar = 500 μm. Lower panel: high magnification. Strong TAZ immunoreactivity was found in the nuclei of fibroblasts that were within the fibroblastic foci and positive for α-SMA (arrow). TAZ staining was negative or weak in the metaplastic epithelial cells (arrowheads) overlying the dense fibrotic lesions. Scale bar = 25 μm.

Next, we investigated TAZ protein expression in lung tissues. Immunohistochemical analysis of normal control lung tissues showed nearly negative immunoreactivity for TAZ in the peripheral lung (Fig. 1a). In contrast, we found strong TAZ immunoreactivity in the nuclei of fibroblasts residing within the fibroblastic foci in IPF lung tissues (Fig. 1b). Importantly, Elastica Masson Goldner (EMG) staining detected abundant collagen fibers in the fibroblastic foci, and TAZ-positive fibroblasts were positively stained for α-SMA, indicating their features of myofibroblasts (Fig. 1b). TAZ staining was negative or weak in metaplastic epithelial cells overlying dense fibrotic lesions (Fig. 1b). Nuclear positivity of TAZ was seen in most of the fibroblastic foci scattered adjacent to fibrotic lesions. These observations were common among the five lung specimens derived from different patients with IPF.

Fibroblastic foci are clusters of fibroblasts and myofibroblasts that represent active fibroproliferative reactions. Distinct nuclear staining for TAZ in the fibroblastic foci suggested that TAZ is active in fibroblasts and plays pathological roles in IPF.

### TAZ is involved in myofibroblast phenotype

To examine the role of TAZ in lung fibroblasts, HFL-1 human lung fibroblasts expressing high levels of endogenous TAZ were used for loss-of-function studies. Preliminary experiments demonstrated that TAZ is located in the nucleus when cells are cultured on plastic tissue culture plates with serum-containing medium. Under these culture conditions, HFL-1 cells expressed different levels of α-SMA, which is a myofibroblastic feature (Supplementary Fig. S2a).

HFL-1 cells were transiently transfected with the negative control (NTC) or TAZ small interfering RNA (siRNA), and silencing of TAZ was confirmed by immunoblotting (Supplementary Fig. S2b). TAZ knockdown in these cells resulted in decreased α-SMA expression, suggesting that TAZ is involved in myofibroblast phenotype (Supplementary Fig. S2c). Transforming growth factor (TGF)-β is an established cytokine to induce α-SMA expression and myofibroblast phenotype^11^. As anticipated, TGF-β stimulation could enhance α-SMA expression in HFL-1 cells with or without TAZ knockdown (Supplementary Fig. S2c).

**Figure 2:**
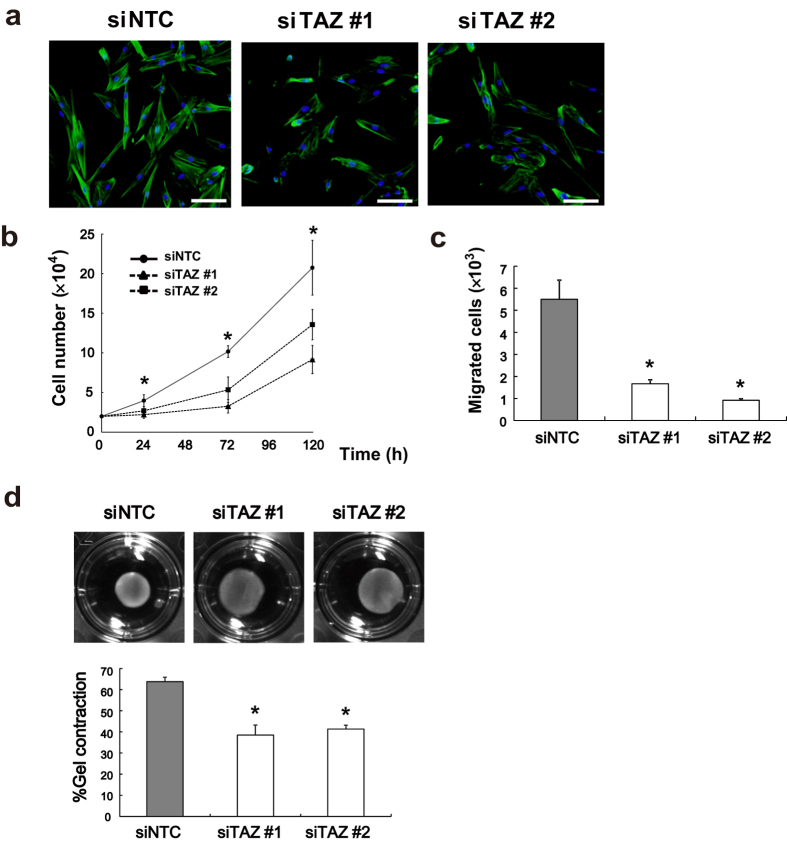
TAZ controls actin stress fiber, proliferation, migration, and collagen gel contraction. (**a**) siRNA-transfected HFL-1 cells were stained with fluorescein-phalloidin (green) to visualize F-actin. DAPI was used for nuclear staining (blue). Scale bar = 100 μm. (**b**) siRNA-transfected HFL-1 cells were seeded at a density of 2 × 10^4^/well in quadruplicate. Cell numbers were counted after 24, 72, and 120 h. (**c**) Migration of siRNA-transfected HFL-1 cells was measured with the Boyden blind-well assay system (n = 8 for each group). Human fibronectin was added to the lower chambers as a chemoattractant. The cells were seeded onto the upper chambers (8 × 10^4^/well) and allowed to migrate for 6 h. The number of migrated cells was determined by the WST-8 assay. (**d**) siRNA-transfected HFL-1 cells were embedded in collagen gels. After 72 h, the area of each gel was quantified. Representative photographs of collagen gels (upper). Percentage of contracted gel area compared to the initial size (lower). Error bars represent standard deviations. ^*^P < 0.05, Student’s *t*-test.

To examine cell morphological changes following TAZ knockdown, we stained the cells with fluorescein-conjugated phalloidin to visualize F-actin. Actin stress fibers were less prominent and less organized in parallel bundles in the cytoplasm of HFL-1 cells transfected with TAZ siRNA (Fig. 2a). These observations implied that actin assembly and formation of stress fibers are closely associated with TAZ expression and myofibroblast phenotype. These findings prompted us to further explore the roles of TAZ in regulating fibroblast function.

### TAZ regulates fibroblast proliferation and migration

It is presumed that the accumulation of fibroblasts precedes deposition of ECM and tissue fibrosis, and two major mechanisms are postulated to be involved. One is expansion of resident fibroblasts and the other is recruitment of fibroblasts from other sites. To test the role of TAZ in these mechanisms, the knockdown effects of TAZ on cell proliferation and migration were investigated.

TAZ knockdown in HFL-1 cells clearly inhibited cell proliferation (Fig. 2b). Next, we evaluated cell migration using the modified Boyden chamber assay with fibronectin as a chemoattractant. HFL-1 cells transfected with TAZ siRNA showed reduced migratory capacity compared to that of control cells (Fig. 2c). In addition, we performed scratch wound healing assay and examined the effect of TAZ knockdown (Supplementary Fig. S3). Consistent with the results of the modified Boyden chamber assay, TAZ knockdown inhibited cell migration in HFL-1 cells. These results were consistent with the notion that TAZ is involved in the accumulation of fibroblasts in the fibroblastic foci.

### TAZ regulates fibroblast-mediated contraction of collagen gel

Three-dimensional collagen gels embedded with fibroblasts are widely used as a cell culture model for fibroblast-mediated tissue contraction^12^. Under these culture conditions, contraction of the collagen gel reflects contraction of fibroblasts and remodeling of collagen. Collagen gels were embedded with siRNA-transfected HFL-1 cells, and gel size was measured. Gels containing TAZ siRNA-transfected cells showed attenuated contraction compared to that of control cells after 72 h (Fig. 2d), indicating that TAZ might contribute to tissue contraction through its effect on lung fibroblasts.

### TAZ is activated on a stiff matrix

**Figure 3:**
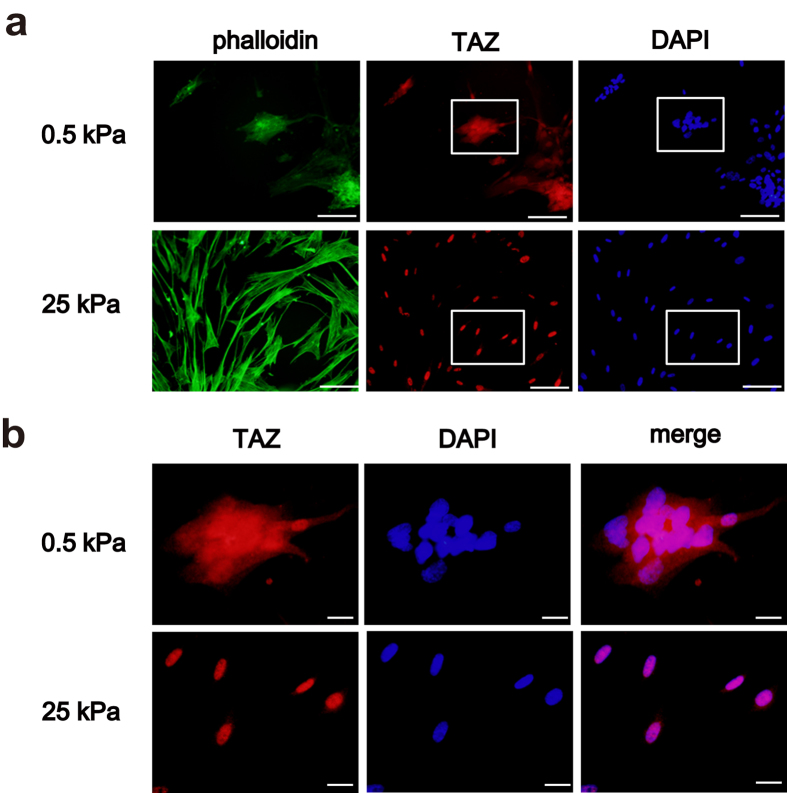
TAZ nuclear localization on a stiff matrix. HFL-1 cells were seeded on type I collagen-coated polyacrylamide hydrogels with different stiffness values (0.5 and 25 kPa). TAZ expression was examined after 48 h by immunofluorescence (red). Cells were also stained with fluorescein-phalloidin (green) to visualize F-actin. DAPI was used for nuclear staining (blue). (**a**) Low magnification. Scale bar = 50 μm. (**b**) High magnification of the area in the white box in (**a**). Scale bar = 10 μm.

ECM remodeling in patients with pulmonary fibrosis leads to tissue stiffness, and stiff matrices impose mechanical stress on cells. The activity of TAZ occurs via nuclear translocation, and subcellular localization of TAZ is dependent on mechanical stress^13^. Thus, we cultured HFL-1 cells on soft and stiff hydrogels coated with type I collagen to assess the effects of matrix stiffness on TAZ subcellular localization in lung fibroblasts. We tested two different stiffness levels corresponding to normal and fibrotic lung tissues (0.5 and 25 kPa, respectively)^14^. Consistent with previous reports, subcellular location of TAZ was sensitive to matrix stiffness. When cultured on a stiff matrix, the cells exhibited stretched morphology, and TAZ was located in the nucleus, which was comparable to the cells cultured on plastic tissue culture dishes (Fig. 3a,b). Notably, TAZ was not stained in the cytoplasm of these cells. In contrast, cells cultured on a soft matrix aggregated into distinct clusters with globular morphology, supporting the idea that matrix stiffness generates mechanical force to facilitate cell stretching. Localization of TAZ in the nucleus was not so prominent, and weak TAZ staining was detected in the cytoplasm (Fig. 3a,b). Furthermore, actin stress fibers visualized by phalloidin staining were more prominent on a stiff matrix (Fig. 3a). Recent studies have demonstrated that stiff matrix promotes cell spreading, and organized F-actin transduces mechanical signals to regulate TAZ nuclear translocation^13^,^15^,^16^,^17^,^18^. Collectively, our findings were in agreement with the notion that matrix stiffness facilitates F-actin organization, mechanotransduction, and TAZ activation. These observations also suggested that TAZ could be preferably activated in fibrotic lesions with stiff matrices, and that activating TAZ might accelerate fibroproliferative reactions.

### Gene expression profiling by RNA-sequencing identifies CTGF as a transcriptional target of TAZ in lung fibroblasts

**Figure 4:**
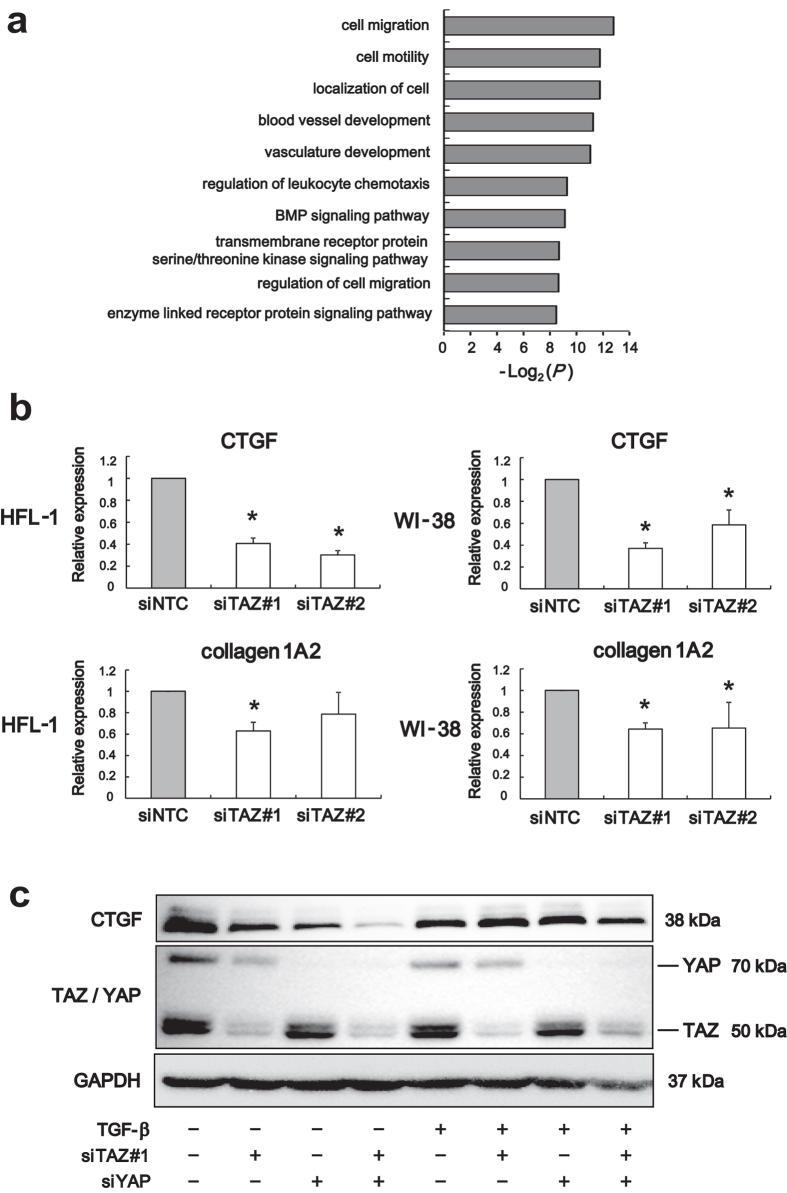
RNA-sequencing identifies connective tissue growth factor (CTGF) as a transcriptional target of TAZ in lung fibroblasts. (**a**) Gene Ontology analysis of the commonly downregulated genes in HFL-1 cells transfected with TAZ siRNA #1 or #2. The data presented is log transformed *P*-values of GO terms enriched in the TAZ-regulated genes. (**b**) Relative expression levels of the indicated transcripts in HFL-1 and WI-38 cells transfected with siNTC or siTAZ were analyzed by qRT-PCR. Error bars represent standard deviations. *P < 0.05, Student’s *t*-test. (**c**) Immunoblotting for TAZ and YAP, and CTGF in HFL-1 cells transfected with TAZ siRNA #1 and/or YAP siRNA in the presence or absence of 5 ng/ml TGF-β stimulation for 48 h. GAPDH was used as the loading control. Molecular weight of each protein is indicated.

To obtain mechanistic insight into the fibrogenic roles of TAZ in lung fibroblasts, we performed RNA sequence-based expression profiling analysis in HFL-1 cells transfected with NTC or TAZ siRNA. We extracted top 600 downregulated genes sorted by false discovery rate (FDR) in HFL-1 cells transfected with TAZ siRNA #1 or #2, and obtained 94 commonly downregulated genes (Supplementary Table S1). Gene Ontology analysis showed that they were enriched with genes involved in cell migration and motility (Fig. 4a). This result was in line with our finding that TAZ regulates migration of lung fibroblasts (Fig. 2c).

Connective tissue growth factor (CTGF) is a member of the CCN family of matricellular proteins, and regulates various biological processes associated with fibrogenesis^19^. CTGF is a well-characterized TAZ target gene in various cell types, and was also downregulated by TAZ knockdown in human lung fibroblasts. Moreover, a component of type I collagen, collagen 1A2, was also included in the genes regulated by TAZ in lung fibroblasts. Downregulation of these genes determined by RNA-sequencing was further validated by quantitative reverse transcription-PCR (qRT-PCR) in HFL-1 and WI-38 lung fibroblasts (Fig. 4b). These findings suggested that the profibrotic roles of TAZ might be mediated, in part, by the expression of CTGF and collagen 1A2.

It is known that TGF-β directly activates CTGF transcription in fibroblasts^20^. Moreover, we have previously demonstrated that TAZ upregulates CTGF, cooperatively with TGF-β signaling in mouse lung epithelial cells^3^. To test whether similar mechanisms are active in human lung fibroblasts, we investigated the effects of TAZ and/or YAP knockdown, and TGF-β stimulation on CTGF expression in HFL-1 cells. TAZ or YAP silencing caused modest suppression of CTGF at protein level while TAZ/YAP double knockdown led to robust CTGF downregulation (Fig. 4c).

### TAZ-regulated genes in human lung fibroblasts

We previously defined TAZ-regulated genes in epithelial or cancer cells by merging the transcriptome data from A549, H441, MCF10A, and SW480 cells^6^. In the present data of lung fibroblasts, TAZ regulated a distinct set of genes that have not been described in other cell types. Indeed, TAZ-regulated genes in lung fibroblasts were associated with cell migration and motility in contrast with our previous observation that TAZ-regulated genes in epithelial or cancer cells are related to cell cycle regulation. By comparing these gene sets, we also noted commonly regulated genes among different cell types, including CTGF, insulin-like growth factor-binding protein 3 (IGFBP3), coronin actin binding protein 1 C (CORO1C), cysteine-rich transmembrane bone morphogenetic protein regulator 1 (CRIM1), and high mobility group nucleosomal binding domain 2 (HMGN2) (Supplementary Fig. S4).

TGF-β is the most potent cytokine to elicit myofibroblast differentiation and enhance production of ECM components, and thereby plays a pivotal role in tissue remodeling^11^. We previously defined TGF-β-regulated genes in lung fibroblasts^21^. When compared to TAZ-regulated genes in lung fibroblasts, 12 genes were common, including IGFBP3, CORO1C, CRIM1, follistatin (FST), follistatin like 1 (FSTL1), and sulfatase 1 (SULF1) (Supplementary Fig. S4). We could confirm enhanced expression of CTGF following TGF-β stimulation (Fig. 4c). However we missed to include CTGF in TGF-β-regulated genes in the previous report, as it failed to reach the cutoff of significant upregulation, possibly due to the technical limitation of microarray^21^. Our observation that TAZ-regulated genes partly overlapped with those regulated by TGF-β, an established fibrogenic factor, further suggested the profibrotic action of TAZ.

### TAZ gene expression and its clinical significance in interstitial lung disease

**Figure 5:**
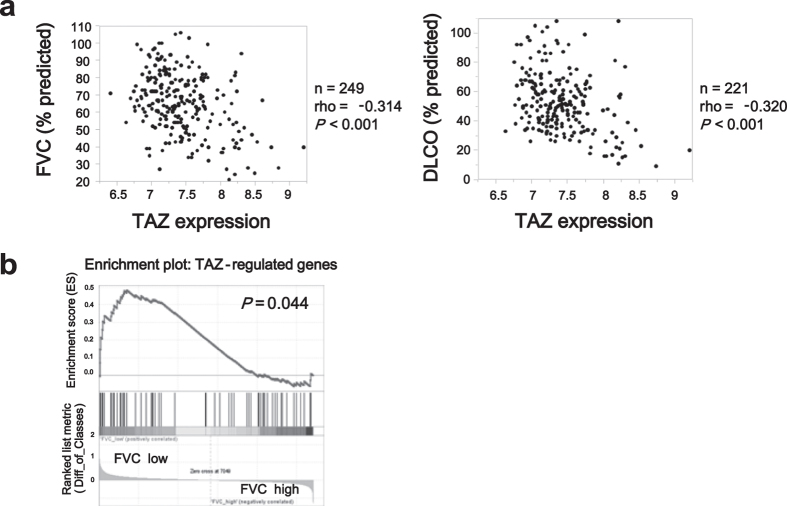
TAZ gene expression and its clinical correlation in patients with interstitial lung disease (ILD). (**a**) Correlation between TAZ expression level and percent predicted forced vital capacity (FVC) or diffusing capacity of the lungs for carbon monoxide (DLCO) in patients with ILD. TAZ expression data were extracted from the mRNA expression catalogue available in the Lung Genomic Research Consortium (LGRC) database (Probe set: A_23_P29769). Details about the samples are available at the LGRC website (https://www.lung-genomics.org). Spearman correlation coefficients (rho) and *P*-values were calculated (n = 249 and 221, respectively). (**b**) Gene set enrichment analysis (GSEA) was performed in the LGRC cohort using the set of 94 TAZ-regulated genes in HFL-1 cells. Patients with ILD were divided into two groups according to percent predicted FVC (cutoff, 50%). The enrichment of TAZ-regulated genes is shown schematically with the genes that correlated best with low FVC on the left and those that correlated best with high FVC on the right.

Finally, we used the Lung Genomics Research Consortium (LGRC) database, which contains gene expression data from lung tissues and lung functions of patients with pulmonary diseases. We investigated the clinical relevance of TAZ in patients with interstitial lung disease (ILD), the most common type of which is IPF. Patients with ILD tend to show reduced forced vital capacity (FVC) and diffusing capacity for carbon monoxide (DLCO), which decline as the disease progresses. We found negative correlation between TAZ expression level and FVC (n = 249, rho = −0.314, *P* < 0.001), and DLCO (n = 221, rho = −0.320, *P* < 0.001) (Fig. 5a). Furthermore, gene set enrichment analysis (GSEA) was performed in the LGRC cohort using the above-defined 94 TAZ-regulated genes in HFL-1 cells. The patients were divided into two groups according to FVC (cutoff, 50% predicted). We confirmed that TAZ-regulated genes were clearly enriched in lung tissues from patients with low FVC (Fig. 5b), suggesting a close relationship between TAZ activation and disease severity.

Next we identified genes differentially expressed in ILD (n = 254) compared to control (n = 108) individuals in the LGRC cohort using a Bioconductor package limma in R. Forty seven genes met the criteria, logFC (fold change) >2 and FDR <10^−4^. When compared to 94 TAZ-regulated genes in lung fibroblasts, only gremlin 1 (GREM1), a bone morphogenetic protein (BMP) antagonist, was overlapped. We confirmed the RNA-sequencing results by qRT-PCR (Supplementary Fig. S5a). Furthermore, positive correlation between TAZ and GREM1 expression levels was found in lung tissues from patients with ILD in the LGRC cohort (Supplementary Fig. S5b). It was noteworthy that Gene Ontology analysis showed enrichment of genes involved in BMP signaling pathway (Fig. 4a), and we noted other BMP accessory proteins such as FST and FSTL among TAZ-regulated genes. Altered expression of BMP accessory proteins in pulmonary fibrosis and their possible role in fibrogenesis has been recently reported^22^. Taken together, the profibrotic action of TAZ may be partially mediated by altered expression of BMP accessory proteins.

## Discussion

A series of recent studies have demonstrated the crucial roles of TAZ/YAP in differentiation and regeneration of lung epithelium^23^,^24^,^25^. However, few studies have considered the roles of TAZ/YAP in lung mesenchymal cells or fibroblasts. Most recently, it has been demonstrated that TAZ/YAP is expressed in the fibroblastic cells of IPF lung tissues, and activated TAZ/YAP drives profibrotic processes in response to mechanical stress^18^. Our findings in the present study augment this previous report and further confirm the functional and clinical significance of TAZ.

We found that TAZ is highly expressed in the fibroblastic foci of lungs from patients with IPF. Cell culture experiments showed that TAZ regulates myofibroblast phenotype, proliferation, migration, and matrix contraction in lung fibroblasts. Importantly, TAZ nuclear localization was more evident when cultured on a stiff matrix, suggesting a TAZ-mediated feed-forward loop of fibroblast activation and tissue fibrosis. Gene expression profiling revealed downstream targets of TAZ, including CTGF and type I collagen. Analyses on the publicly available transcriptome data further substantiated the clinical impact of TAZ.

Evidence is accumulating that suggests crosstalk between TAZ and other signaling pathways such as TGF-β/Smad. In response to TGF-β stimulation, TAZ forms complexes with Smad2/3 that translocate into the nucleus and regulate target gene transcription^26^. Because TGF-β/Smad signaling pathway is important in the pathogenesis of IPF, TAZ may play crucial roles as a hub to integrate mechanotransduction and TGF-β signaling in the context of lung fibrosis^27^.

This study was the first to perform gene expression profiling following TAZ knockdown in human lung fibroblasts, and CTGF was identified to be controlled by TAZ. As CTGF is implicated in lung fibroblast proliferation, migration, and ECM synthesis, the fibrogenic effects of TAZ could, in part, be mediated by CTGF^28^,^29^. Interestingly, previous reports have demonstrated that CTGF is cooperatively regulated by TAZ/YAP and Smad2/3^30^. In the present study, comparison between TAZ-regulated and TGF-β-regulated genes in lung fibroblasts revealed a subset of common genes that could be coordinately regulated. Further studies may reveal their potential involvement in fibrogenic reactions or usefulness as molecular markers.

Most recently, it has been reported that YAP regulates differentiation of dermal fibroblasts into highly contractile myofibroblasts, and that YAP expression levels and nuclear localization are elevated in affected tissues from patients with Dupuytren disease, a fibroproliferative disorder of the hands and fingers^31^. Thus, it would be worthy of investigation whether the profibrotic functions of TAZ observed in this study are also involved in a variety of other fibrotic disorders.

Although the antifibrotic agent pirfenidone and the multi-targeted tyrosine kinase inhibitor nintedanib have been approved for clinical use in some countries, patients with IPF still have poor prognosis^32^,^33^. A high-throughput screening study identified verteporfin as a small molecule inhibitor of interactions between YAP and TEAD^34^. Additionally, Rho and Rho-associated protein kinase (ROCK) inhibitors suppress TAZ/YAP nuclear localization and activity^13^. Given that TAZ/YAP plays fibrogenic roles, such pharmacological inhibitors may be beneficial for treating IPF. Indeed, previous studies suggested that pulmonary fibrosis could be treated by Rho and ROCK inhibition^35^. It has been demonstrated that pulmonary radiation injury and bleomycin-induced pulmonary fibrosis were successfully modulated using Rho and ROCK inhibitors (statins and Y-27632)^36^.

In summary, we elucidated the functional role of TAZ in pulmonary fibrosis, through an integrated analysis that employs bioinformatics, cell cultures, and clinical samples. Our study reveals a mechanism of sustained activation of lung fibroblasts that contributes to pulmonary fibrosis, and offers a potential basis for therapeutic intervention.

## Methods

### Cell culture

HFL-1 and WI-38 human lung fibroblasts were purchased from the American Type Culture Collection (Rockville, MD, USA) and cultured in Dulbecco’s Modified Eagle’s Medium (DMEM) supplemented with 10% fetal bovine serum (FBS) on plastic tissue culture dishes. TGF-β1 was purchased from R&D Systems (Minneapolis, MN, USA) and was used at the concentration of 5 ng/ml. To assess the effects of matrix stiffness on TAZ activity, HFL-1 cells were seeded on type I collagen-coated polyacrylamide hydrogels with different stiffness values (Matrigen Life Technologies, Brea, CA, USA). For cell proliferation analysis, HFL-1 cells were seeded in quadruplicate at 2 × 10^4^/well in a 12-well plate. Cell numbers were counted after 24, 72, and 120 h.

### Small interfering RNA experiments

siRNA against human TAZ (Stealth RNAi, siTAZ #1: HSS119546; siTAZ #2: HSS119547), YAP (Stealth RNAi, HSS173621), and the negative control (Stealth RNAi, siNTC: #12935-300) were purchased from Invitrogen (Carlsbad, CA, USA). The cells were transfected with 20 nM siRNA using Lipofectamine RNAiMAX (Invitrogen) according to the manufacturer’s instructions.

### Cell migration analysis

Cell migration was analyzed using the modified Boyden chamber assay with a 96-well chemotaxis chamber and a membrane with 8 μm pore size (ChemoTx, 116 series; Neuro Probe, Gaithersburg, MD, USA). siRNA-transfected HFL-1 cells were suspended in serum-free DMEM, and 80 μL of cell suspension (8 × 10^4^/well) was seeded onto the filters in the upper chambers. The lower chambers were filled with DMEM supplemented with 5 μg/mL fibronectin (Sigma-Aldrich, St. Louis, MO, USA) as a chemoattractant. The cells were allowed to migrate from the upper to the lower chambers through the filters for 6 h. The number of migrated cells was determined by the WST-8 assay (Nacalai Tesque, Kyoto, Japan). The mean of eight chambers was calculated for each group.

For scratch wound healing assay, HFL-1 cells were cultured at confluency, and wounds were generated in the central area of each well. Photographs were taken immediately after the scratch and after 24 h. The distance of cell migration was measured under phase-contrast microscopy.

### Collagen gel contraction assay

Three-dimensional gel cultures were carried out according to the protocol published previously^37^. Briefly, collagen gels were prepared by mixing 0.5 mL of fibroblast cell suspension (1 × 10^6^ cells) in FBS, 2.3 mL type I collagen (Cell matrix type IA; Nitta Gelatin, Tokyo, Japan), 670 μL of 5 × DMEM, and 330 μL reconstitution buffer. The mixture (3 mL) was cast into each well of a 6-well plate. The solution was allowed to polymerize at 37 °C for 30 min. After overnight incubation, each gel was detached and cultured in serum-free DMEM. After 72 h, the surface area of the gels was quantified.

### Quantitative reverse transcription-PCR (qRT-PCR)

Total RNA was extracted from the cells using an RNeasy Mini Kit (Qiagen, Valencia, CA, USA), and first-strand cDNA was synthesized using SuperScript III Reverse Transcriptase (Invitrogen). The qRT-PCR analysis was performed using the Mx-3000P qPCR System (Stratagene, La Jolla, CA, USA) and QuantiTect SYBR Green PCR (Qiagen) according to the manufacturer’s protocol. Expression levels were normalized to that of glyceraldehyde-3-phosphate dehydrogenase (GAPDH). The PCR primers used are as follows: TAZ forward, 5′-GTA TCC CAG CCA AAT CTC GTG ATG-3′; TAZ reverse, 5′-CAG CGC ATT GGG CAT ACT CAT G -3′; CTGF forward, 5′-CTT GCG AAG CTG ACC TGG AA-3′; CTGF reverse, 5′-AAA GCT CAA ACT TGA TAG GCT TGG A-3′; collagen 1A2 forward, 5′-GAA AAC ATC CCA GCC AAG AA-3′; collagen 1A2 reverse, 5′-GCC AGT CTC CTC ATC CAT GT-3′; GAPDH forward, 5′-GGT GAA GGT CGG AGT CAA CGG A-3′; GAPDH reverse, 5′-GAG GGA TCT CGC TCC TGG AAG A-3′; GREM1 forward, 5′-AAG CGA GAC TGG TGC AAA AC-3′; GREM1 reverse, 5′-CTT GCA GAA GGA GCA GGA CT-3′.

### RNA sequencing

Total RNA was extracted from HFL-1 cells 48 h after NTC or TAZ siRNA transfection and sequenced using Ion Proton (Ion Torrent, Guilford, CT, USA). Two different TAZ siRNA sequences were transfected in duplicate. We used the following external applications for bioinformatics: FastQ generation (Ion Plugin ver. 4.0.37–1), BAM Alignment to Hg19 (TMAP ver. 3.4.1, the Torrent Mapping Alignment Program for Ion Torrent Data), and Read count analysis (HTseq ver. 0.6.1, Ensemble annotation). Datasets are deposited in the Gene Expression Omnibus (GEO) database (http://www.ncbi.nlm.nih.gov/geo) (GSE73555). We assessed the downregulated genes after TAZ knockdown using EdgeR in R ver. 3.1.0. We extracted the top 600 downregulated genes, which were determined by FDR in HFL-1 cells transfected with TAZ siRNA #1 or #2, and isolated 94 commonly downregulated genes. Gene Ontology analysis of these genes was performed using the DAVID 6.7 functional annotation tool^38^. Gene set enrichment analysis was performed as described previously^39^.

### Immunoblot analysis

Cells were lysed, followed by SDS gel electrophoresis and semi-dry transfer of the proteins to polyvinylidene difluoride membranes, which were probed with anti-TAZ (#2149; Cell Signaling Technology, Beverly, MA, USA). anti-TAZ/YAP (#8418; D24E4, Cell Signaling Technology), anti-CTGF (sc14939; Santa Cruz Biotechnology, Dallas, TX, USA), or anti-α-SMA (Sigma-Aldrich) antibodies, and the appropriate horseradish peroxidase-conjugated secondary antibodies. Immunodetection was performed with the ECL Prime Western Blotting Detection Kit (GE Healthcare, Buckinghamshire, UK).

### Immunofluorescence

Cells were fixed in formalin, permeabilized with 0.3% Triton X-100, and blocked with 5% normal goat serum in PBS for 1 h. The cells were incubated with anti-TAZ antibody (1:250 dilution, HPA007415; Sigma-Aldrich) or anti-α-SMA (1:250 dilution, Sigma-Aldrich) at 4 °C overnight, and further incubated for 1 h with secondary goat anti-rabbit Alexa Fluor 594-conjugated antibody (1:500 dilution, Life Technologies) or anti-mouse Alexa Fluor 488-conjugated antibody (1:500 dilution, Life Technologies), respectively. siRNA-transfected HFL-1 cells were stained with Acti-stain 488 phalloidin to detect F-actin (Cytoskeleton Inc., Denver, CO, USA), and the nuclei were stained with DAPI.

### Immunohistochemistry

Lung specimens of pulmonary fibrosis were obtained from five patients pathologically diagnosed with usual interstitial pneumonia (UIP). Normal control lung tissues were obtained from normal areas of surgically resected lungs from patients with lung cancer. The study protocol was approved by the Human Ethics Review Committee of Nippon Medical School and informed consent was obtained from all patients. Paraffin sections were deparaffinized on silane-coated slides, rehydrated and immersed in a solution of 0.3% hydrogen peroxide in methanol for 30 min to eliminate endogenous peroxidase. The tissue sections were processed for epitope retrieval by heat (120 °C, 20 min) in 0.01 M sodium citrate buffer (pH 6.0) and incubated with anti-TAZ (1:100 dilution, Sigma-Aldrich) or anti-α-SMA (1:500 dilution, Dako, Glostrup, Denmark) antibodies at 4 °C overnight. The sections were then incubated with Histofine Simple Stain Kits (Nichirei Biosciences, Tokyo, Japan) as secondary antibodies with peroxidase for 30 min. Peroxidase activity was detected with a solution of 3,3′-diaminobenzidine and H_2_O_2_, and counterstained with Mayer’s hematoxylin.

### Statistical analysis

Differences were detected with Student’s *t-*test using JMP Pro ver. 11.0.0 software (SAS Institute, Cary, NC, USA). Spearman’s rho was used to quantify the correlation between TAZ expression level and clinical parameters. *P* < 0.05 was considered significant. All data are expressed as means ± standard deviations.

## Additional Information

**How to cite this article**: Noguchi, S. *et al*. TAZ contributes to pulmonary fibrosis by activating profibrotic functions of lung fibroblasts. *Sci. Rep.* **7**, 42595; doi: 10.1038/srep42595 (2017).

**Publisher's note:** Springer Nature remains neutral with regard to jurisdictional claims in published maps and institutional affiliations.

## Supplementary Material

Supplementary File
