# Supplementary material for: TAZ contributes to pulmonary fibrosis by activating profibrotic functions of lung fibroblasts
Source: Sci Rep. 2017 Feb 14;7:42595. doi: 10.1038/srep42595 (PMC5307361; doi:10.1038/srep42595)

TAZ contributes to pulmonary fibrosis by activating profibrotic functions of lung fibroblasts

Satoshi Noguchi[1], Akira Saito[1, 2*], Yu Mikami[1, 3], Hirokazu Urushiyama[1, 4], Masafumi Horie[1, 2], Hirotaka Matsuzaki[1], Hideyuki Takeshima[1], Kosuke Makita[1], Naoya Miyashita[1], Akihisa Mitani[1], Taisuke Jo[1, 2], Yasuhiro Yamauchi[1], Yasuhiro Terasaki[4] and Takahide Nagase[1]

Supplementary Information

Supplementary figure legends

Supplementary Figure S1. TAZ expression in lung fibroblasts and epithelial cells.

(**a**) TAZ expression in lung fibroblasts and epithelial cells. Cap analysis gene expression (CAGE) data were obtained from the ZENBU database (http://fantom.gsc.riken.jp/zenbu/). Y-axis represents CAGE tag counts (tags per million: TPM). SAECs, Small airway epithelial cells; AECs, Alveolar epithelial cells. (**b**) TAZ expression in lung fibroblasts derived from normal or idiopathic pulmonary fibrosis (IPF) lungs was analyzed using the GSE40839 microarray dataset. Probe set 202132_at. was used. *$P < 0.05$, Student's *t* -test.

Supplementary Figure S2. TAZ knockdown and myofibroblast phenotype in HFL-1 cells cultured on plastic tissue culture plates.

(**a**) TAZ and α-smooth muscle actin (α-SMA) expression was examined by immunofluorescence in HFL-1 cells seeded on plastic tissue culture plates (red and green, respectively). DAPI was used for nuclear staining (blue). Scale bar = 50 μm. (**b**) HFL-1 cells were transfected with siNTC (control siRNA) or siTAZ (TAZ siRNA #1 and #2). Immunoblotting for TAZ was performed. GAPDH was used as the loading control. Molecular weight of each protein is indicated. (**c**) HFL-1 cells were transfected with siNTC (control siRNA) or siTAZ (TAZ siRNA #1 and #2) in the presence or absence of 5 ng/ml TGF-β stimulation. Immunoblotting for α-SMA was performed.

GAPDH was used as the loading control. Molecular weight of each protein is indicated.

Supplementary Figure S3. Scratch wound healing assay.

(**a**) HFL-1 cells transfected with siNTC (control siRNA) or siTAZ (TAZ siRNA #1 and #2) were grown at confluency, and wounds were generated by cell scratching. The distance of cell migration was measured after 24 h for each group. (**b**) Relative distance of cell migration compared to the control is shown (n = 3 for each group). Error bars represent standard deviations. *$P < 0.05$, Student's *t*-test.

Supplementary Figure S4. Venn diagram showing overlaps of TAZ-regulated genes in lung fibroblasts, those in epithelial or cancer cells, and transforming growth factor (TGF)-β-regulated genes in lung fibroblasts. The number in parentheses represents the total number of genes in each group.

Supplementary Figure S5. TAZ regulates the expression of gremlin 1.

(**a**) Relative expression levels of gremlin 1 (GREM1) in HFL-1 cells transfected with siNTC or siTAZ were analyzed by qRT-PCR. Error bars represent standard deviations. *$P < 0.05$, Student's *t*-test. (**b**) Correlation between TAZ and GREM1 expression was examined in lung tissues from patients with interstitial lung disease (ILD) in the Lung Genomics Research Consortium (LGRC) cohort. Spearman correlation coefficients (rho) and the *P* value were calculated.

Supplementary Table S1. The commonly downregulated genes in HFL-1 cells transfected with TAZ siRNA #1 or #2.

Gene symbol	Gene name
ABCF1	ATP-binding cassette, sub-family F (GCN20), member 1
ABHD2	abhydrolase domain containing 2
ACSS1	acyl-CoA synthetase short-chain family member 1
ADAMTS15	ADAM metallopeptidase with thrombospondin type 1 motif, 15
ADH1B	alcohol dehydrogenase 1B (class I)
ADM2	adrenomedullin 2
AHNAK	AHNAK nucleoprotein
ALDH1L2	aldehyde dehydrogenase 1 family, member L2
ALDH6A1	aldehyde dehydrogenase 6 family, member A1
ANKRD13A	ankyrin repeat domain 13A
AOX1	aldehyde oxidase 1
ARID5B	AT rich interactive domain 5B (MRF1-like)
ARL6IP1	ADP-ribosylation factor-like 6 interacting protein 1
ASNS	asparagine synthetase
ATP2B4	ATPase, Ca++ transporting, plasma membrane 4
AURKAPS1	aurora kinase A pseudogene 1
B3GALT2	beta 1,3-galactosyltransferase, polypeptide 2
BCYRN1	brain cytoplasmic RNA 1 (non-protein coding)
C20orf194	chromosome 20 open reading frame 194
CAV1	caveolin 1, caveolae protein, 22kDa
CBX5	chromobox homolog 5 (HP1 alpha homolog, Drosophila)
CCBE1	collagen and calcium binding EGF domains 1
CDC25B	cell division cycle 25 homolog B (S. pombe)
CELF2	CUG triplet repeat, RNA binding protein 2
CHSY1	chondroitin sulfate synthase 1
COL1A2	collagen, type I, alpha 2
CORO1C	coronin, actin binding protein, 1C
COX15	COX15 homolog, cytochrome c oxidase assembly protein (yeast)
CRIM1	cysteine rich transmembrane BMP regulator 1 (chordin-like)
CTGF	connective tissue growth factor

CXCL12	chemokine (C-X-C motif) ligand 12 (stromal cell-derived factor 1)
DAB2	disabled homolog 2, mitogen-responsive phosphoprotein (Drosophila)
DDIT4	DNA-damage-inducible transcript 4
DOCK5	dedicator of cytokinesis 5
EIF1AX	eukaryotic translation initiation factor 1A, X-linked
ELL2	elongation factor, RNA polymerase II, 2
ELOVL2	elongation of very long chain fatty acids (FEN1/Elo2, SUR4/Elo3, yeast)-like 2
EMG1	EMG1 nucleolar protein homolog (S. cerevisiae)
FAM107B	family with sequence similarity 107, member B
FOXM1	forkhead box M1
FST	follistatin
FSTL1	follistatin-like 1
GARS	glycyl-tRNA synthetase
GAS5	growth arrest-specific 5 (non-protein coding)
GDAP1	ganglioside-induced differentiation-associated protein 1
GFRA1	GDNF family receptor alpha 1
GREM1	gremlin 1, cysteine knot superfamily, homolog (Xenopus laevis)
GRPR	gastrin-releasing peptide receptor
GUCY1A2	guanylate cyclase 1, soluble, alpha 2
GUCY1B3	guanylate cyclase 1, soluble, beta 3
HADHA	hydroxyacyl-Coenzyme A dehydrogenase/3-ketoacyl-Coenzyme A thiolase/enoyl-Coenzyme A hydratase (trifunctional protein), alpha subunit
HDDC3	HD domain containing 3
HEBP1	heme binding protein 1
HIST1H2BK	histone cluster 1, H2bk
HMGN2	high-mobility group nucleosomal binding domain 2
HSPB6	heat shock protein, alpha-crystallin-related, B6
IGFBP3	insulin-like growth factor binding protein 3
IL6R	interleukin 6 receptor
IMPDH2	IMP (inosine monophosphate) dehydrogenase 2
INHBE	inhibin, beta E
KCNE4	potassium voltage-gated channel, Isk-related family, member 4
KCNJ2	potassium inwardly-rectifying channel, subfamily J, member 2
KCNN2	potassium intermediate/small conductance calcium-activated channel, subfamily N, member 2

KIAA1644	KIAA1644
MASP1	mannan-binding lectin serine peptidase 1 (C4/C2 activating component of Ra-reactive factor)
MATN2	matrilin 2
MICALL1	MICAL-like 1
MOV10	Mov10, Moloney leukemia virus 10, homolog (mouse)
NAV2	neuron navigator 2
PCK2	phosphoenolpyruvate carboxykinase 2 (mitochondrial)
PHACTR4	phosphatase and actin regulator 4
PMP22	peripheral myelin protein 22
PNPO	pyridoxamine 5'-phosphate oxidase
PPAP2B	phosphatidic acid phosphatase type 2B
PPP1R3C	protein phosphatase 1, regulatory (inhibitor) subunit 3C
PRSS23	protease, serine, 23
RBMS2	RNA binding motif, single stranded interacting protein 2
RELN	reelin
RGMB	RGM domain family, member B
RLIM	ring finger protein, LIM domain interacting
RPL23P8	ribosomal protein L23 pseudogene 8
S100A10	S100 calcium binding protein A10
SH3D19	SH3 domain containing 19
SLC7A14	solute carrier family 7 (cationic amino acid transporter, y+ system), member 14
SLC7A5	solute carrier family 7 (cationic amino acid transporter, y+ system), member 5
SULF1	sulfatase 1
TGFBR3	transforming growth factor, beta receptor III
TMBIM6	transmembrane BAX inhibitor motif containing 6
TRIM25	tripartite motif-containing 25
TXNIP	thioredoxin interacting protein
WASF2	WAS protein family, member 2
WNT2	wingless-type MMTV integration site family member 2
WWTR1	WW domain containing transcription regulator 1
XBP1	X-box binding protein 1

Supplementary Figure S1

a

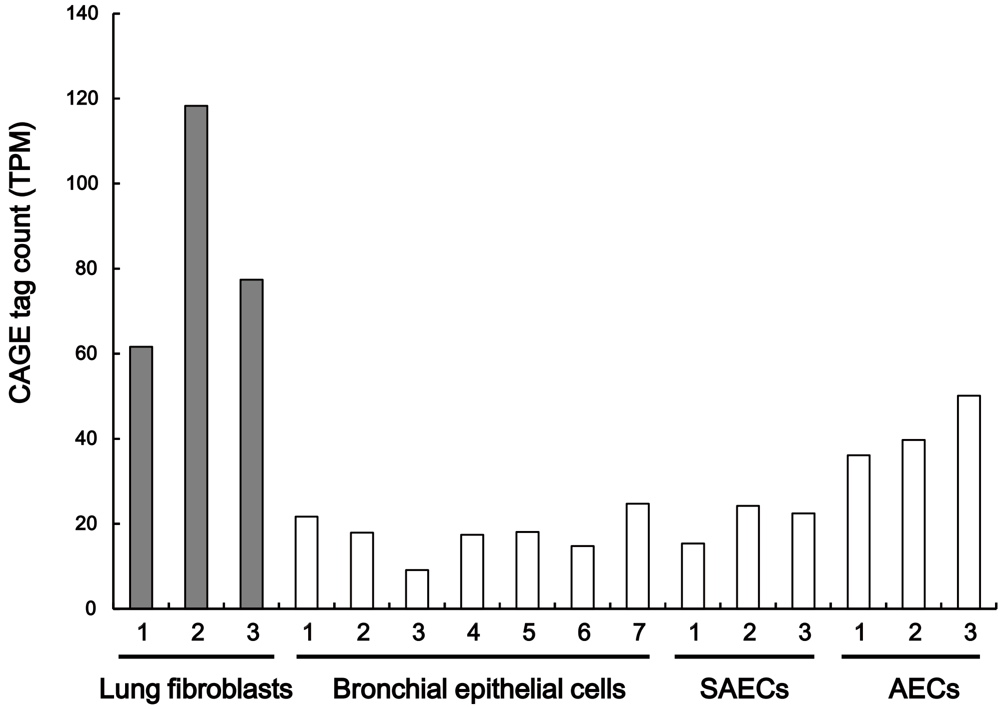

b

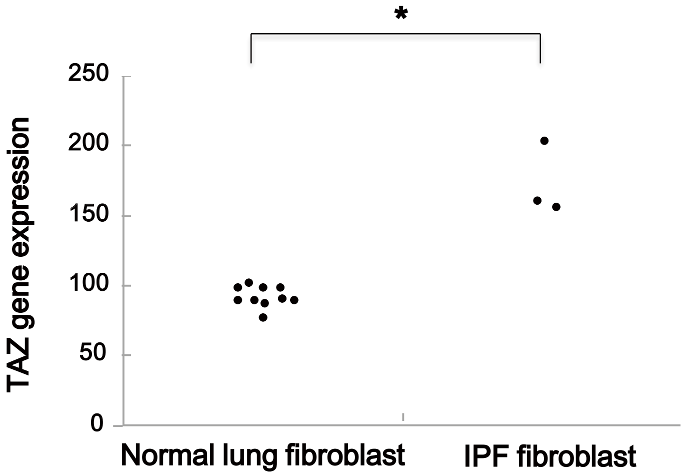

Supplementary Figure S2

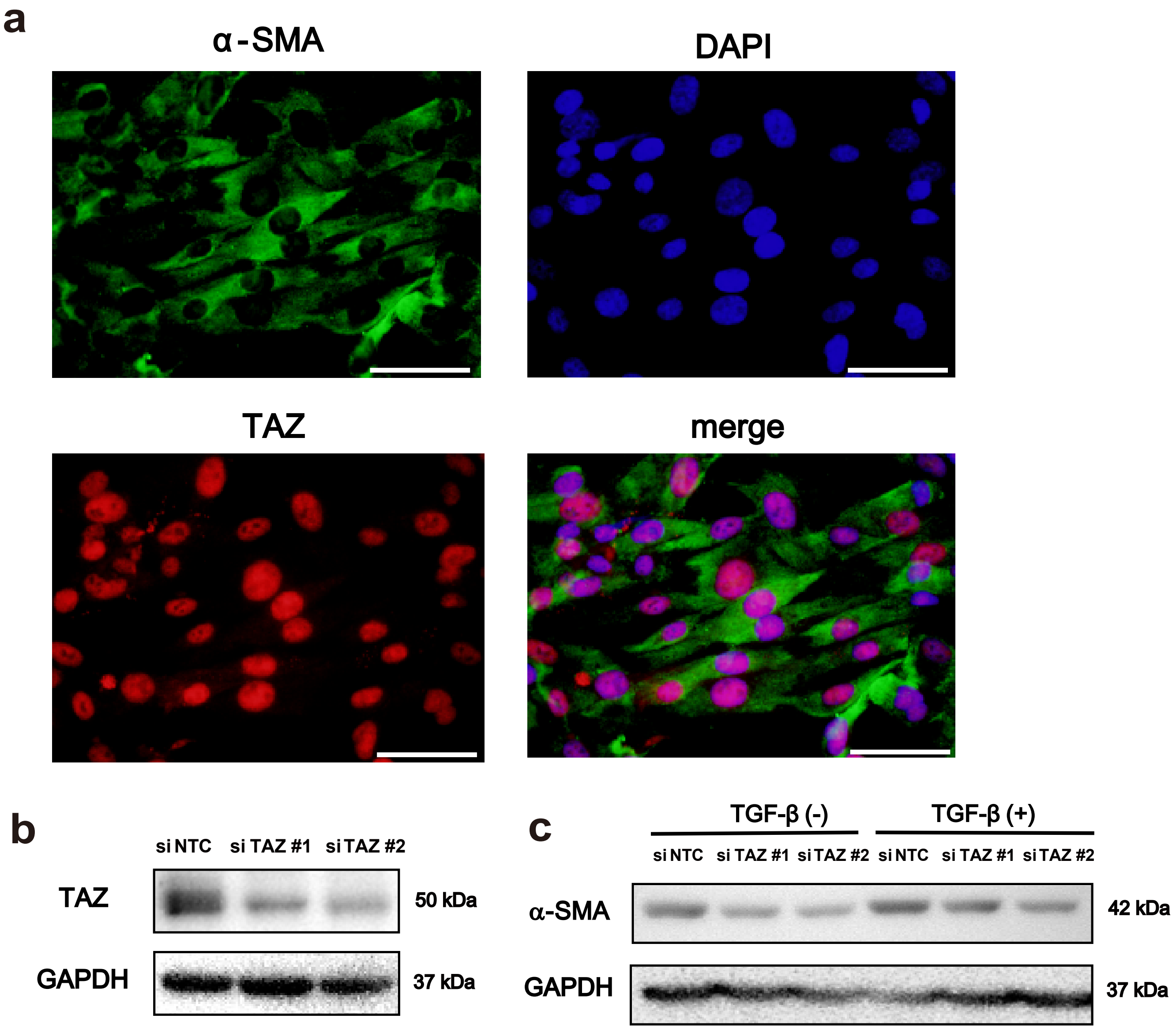

Supplementary Figure S3

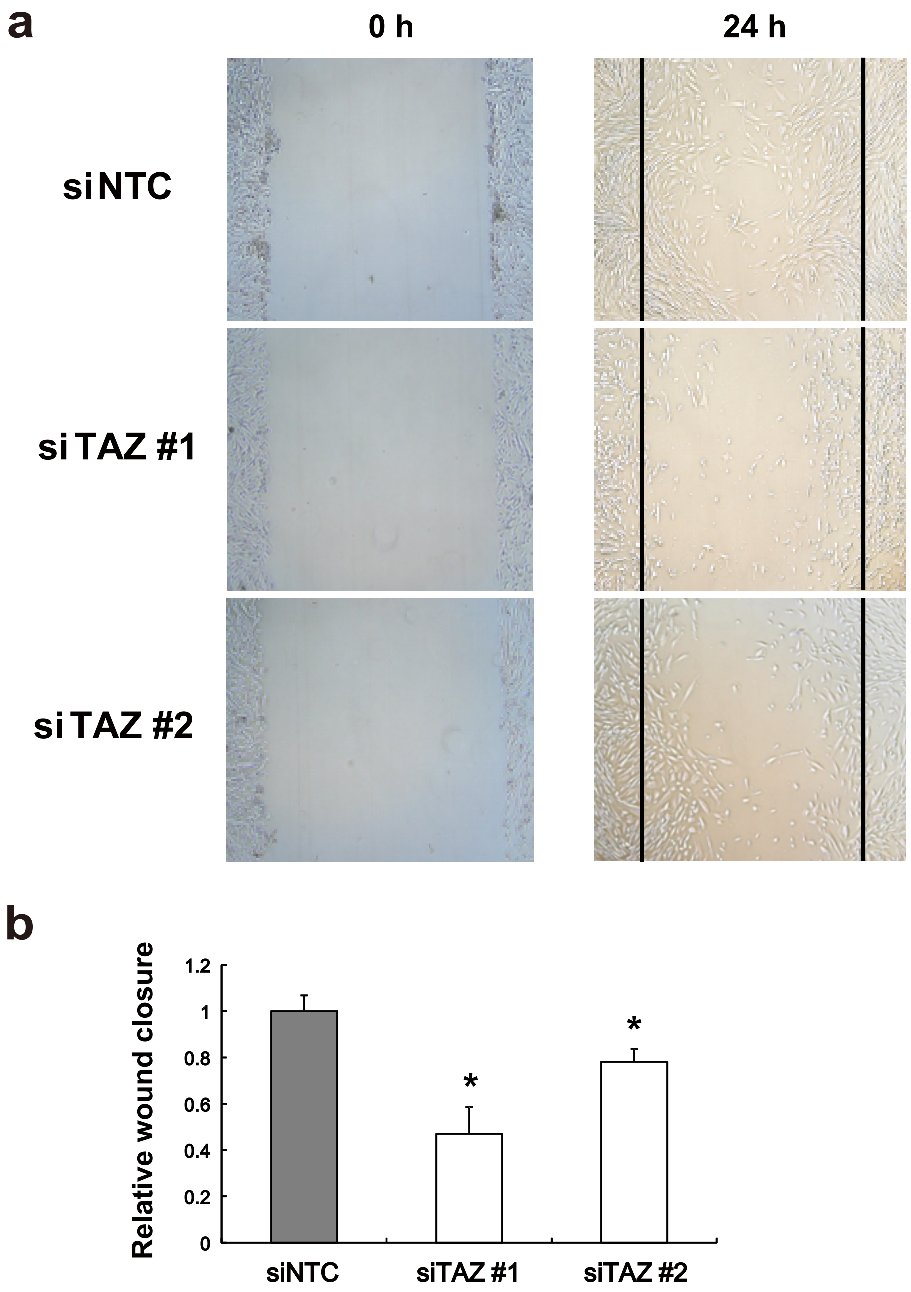

Supplementary Figure S4

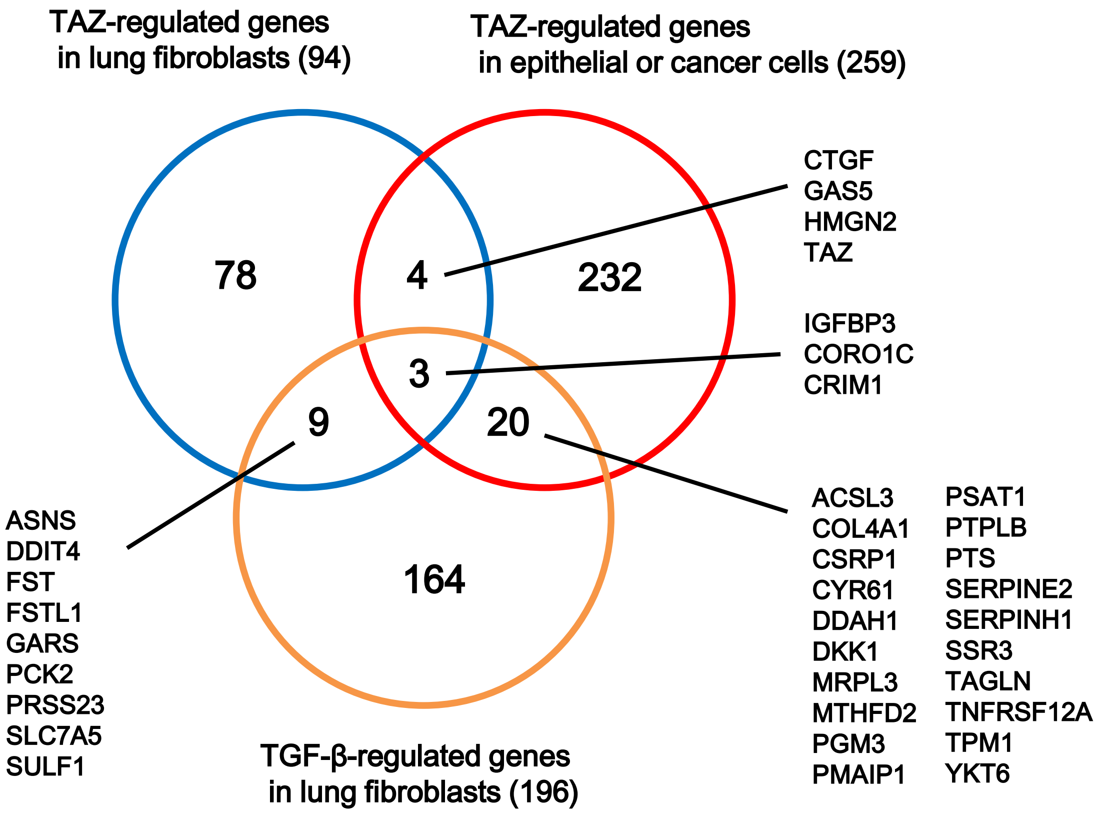

Supplementary Figure S5

a

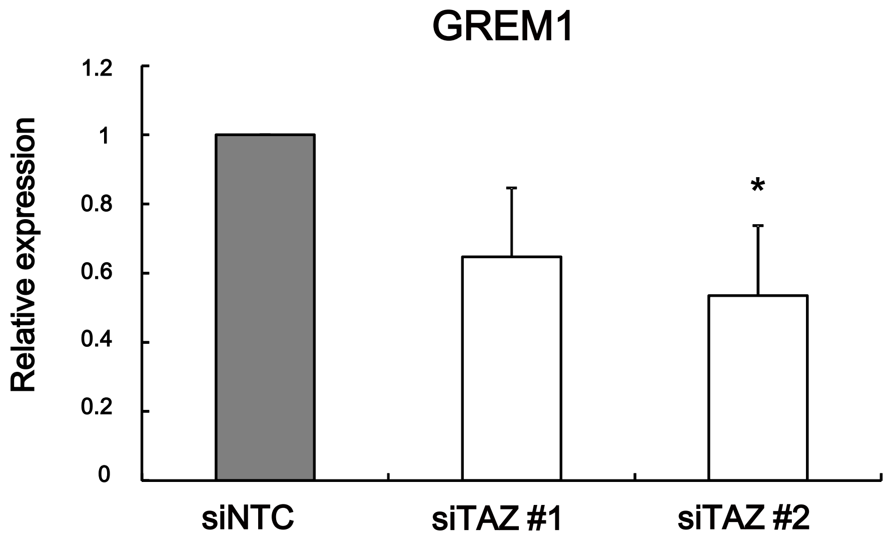

b

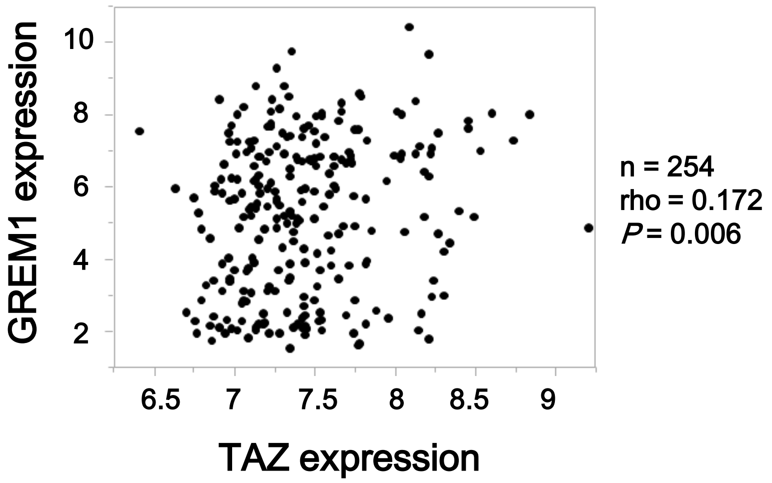

Supplement: Supplementary File [file srep42595-s1.pdf]
